# Nursing home geriatric rehabilitation care and interprofessional collaboration; a practice-based study

**DOI:** 10.1186/s12877-023-04212-6

**Published:** 2023-09-05

**Authors:** Hans Drenth, Wim Krijnen, Lourens van der Weerd, Jan Jaap Reinders, Hans Hobbelen

**Affiliations:** 1 Research group Healthy Ageing, Allied Health Care and Nursing, Hanze University of Applied Sciences, Groningen, The Netherlands; 2 ZuidOostZorg, Friesland, The Netherlands; 3 FAITH research, Leeuwarden, Groningen, The Netherlands; 4 LEARN, Research Institute SHARE, Groningen, University of Groningen, University Medical Center Groningen, Groningen, The Netherlands; 5 Research Group on Interprofessional Identity and Collaboration, Kaunas University of Applied Sciences, Kaunas, Lithuania; 6 Department of General Practice and Elderly Care Medicine, University Medical Center Groningen, University of Groningen, Groningen, the Netherlands

**Keywords:** Geriatric rahabilitation, Interprofessional collaboration, Interprofessional identity

## Abstract

**Background:**

Frailty and multimorbidity are common among patients in geriatric rehabilitation care (GRC). Proper care of these patients involves multiple professionals which requires optimal interprofessional collaboration to provide the best possible support. Interprofessional collaboration (IPC) goes beyond multi-professional collaboration. It requires professionals to think beyond the expertise of their own discipline and work on joint outcomes in which the patient is actively involved. This study describes the development of the GRC teams of an elderly care organization towards the IPC.

**Methods:**

Mixed method pre-post study of 15 months. The interprofessional training program comprised team trainings, webinars, and online team sessions. Data was aggregated by administering the Extended Professional Identity Scale (EPIS) and QuickScan Interprofessional Collaboration (QS) measurements to GRC staff and by observations of the multi-professional team consultation (MPC) meetings of six GRC teams of an organization for elderly care in Drachten and Dokkum in the Netherlands. ADL independence (Barthel Index) and number of inpatient days were analyzed before and after the project.

**Results:**

Pretest healthcare professional response was 106, patients for analyses was 181; posttest response was 84, patients was 170. The EPIS shows improvement on “interprofessional belonging” (P = .001, 95%CI: 0.57–2.21), “interprofessional commitment” (P = .027, 95%CI: 0.12–1.90), and overall “interprofessional identity” (P = .013, 95%CI: 0.62 − 5.20). On the QS, all domains improved; “shared values” (P = .009, 95%CI: 0.07 − 0.47), “context” (P = .005, 95%CI: 0.08 − 0.44), “structure & organization” (P = .001, 95%CI: 0.14 − 0.56), “group dynamics & interaction” (P < .001, 95%CI: 0.18 − 0.58), and “entrepreneurship & management” (P = .039, 95%CI: 0.01 − 0.48). A qualitative analysis of the reflection responses and MPC observations indicate a shift from multi-professional to more IPC. Differences in ADL over time were not statistically significant. The mean number of inpatient days was reduced by 11.8 (P < .001, 95%CI: -17.34 - − 6.31) days.

**Conclusions:**

Within the GRC teams, there was a shift observed to more IPC and better representation of the patient’s wishes and needs. ADL independence did not change, yet we found a statistically significant decrease in the number of inpatient days. The basis for IPC was well established, however, it remains necessary that the teams continue to develop and invest in the collaboration with each other and the patient to further improve it.

**Supplementary Information:**

The online version contains supplementary material available at 10.1186/s12877-023-04212-6.

## Background

In the coming years, the number of older people in the Netherlands will increase, and individuals will also be older. This is commonly accompanied by frailty and multiple chronic conditions (multi-morbidity), making geriatric rehabilitation care (GRC) increasingly complex within nursing homes [1–3]. The comprehensive care of these patients is organized in GRC teams involving multiple healthcare professionals with different specializations, which requires optimal collaboration to provide the best care possible [4–6].

Interprofessional collaboration (IPC) is a way of working together that goes beyond multi-professional collaboration and is in accordance with trends and developments in healthcare that can further improve the quality of care [7]. It is a form of collaboration in which several health professionals with different professional backgrounds offer care, treatment, guidance, and support to patients. In this process, the patient and his social network – if they are willing and able at the time – are actively involved in the entire process of identification, diagnosis, choice of treatment, the treatment itself, monitoring aftercare, and evaluation in order to provide the highest quality of care within different contexts [8]. Multi-professional collaboration denotes that several professionals are active around the patient, yet each is acting within its own expert boundaries and focused on its own professional outcomes [7]. In addition, the interaction between the different disciplines is limited [9]. IPC is about providing care as a team, looking integrally at the patient, and working on a joint outcome without neglecting the specific competences of each discipline [7].

In the GRC, three themes are identified that can be considered as promoting or hindering factors for IPC, i.e., team collaboration, information sharing, and organizational factors[10]. For IPC, however, it is important that team members also have the intrinsic motivation to do so of which a specific form is identification with a group. This “group identity” (or “social identity”) refers to a psychological association of an individual with a group that has its own theme [11, 12]. In this context, an “interprofessional identity (IPI)” is a social identity with an “identity theme” that relates to IPC and to a group larger than an individual’s own profession [13]. An IPI can be regarded as identification with an interprofessional team role that consists of a sense of membership, an emotional relationship with the identity group, and an orientation towards IPC [14]. IPI can therefore be divided into the following domains: Interprofessional belonging, interprofessional commitment, and interprofessional beliefs [13]. Effective IPC begins with personal, social, and professional acquaintances; appreciation of the added value of the other professionals; and recognition of the importance of the interdependence of team members [15]. Because of widespread monodisciplinary education, many professionals are not accustomed to functioning beyond their role boundaries and have their own professional communication[16, 17] To truly achieve IPC, it is important to reflect on personal factors, roles, and responsibilities of the team and team members. To achieve this, interprofessional education (including on-the-job training) and (inter)professional change of behavior is important [18].

To develop the IPC within the GRC teams of a healthcare organization for older people in the Netherlands, an interprofessional training program on IPC education and team collaboration has been initiated. The objective of this study is to determine whether this program has led to IPC and changed interprofessional identity as well as whether there is an impact on the quality of care.

## Methods

### Design

Mixed method: Quantitative and qualitative pre-posttest study with a follow up of 15 months.

### Participants

Healthcare professionals from six GRC teams in Drachten and Dokkum in the Netherlands. A GRC team consists of a nursing staff (NS) of 13–17 (range) secondary vocational and higher professional nurses and 8–16 (range) treatment staff (TS) members (elderly care physician, clinical nurse specialist, occupational therapist, physical therapist, dietician, speech therapist, social worker, psychologist). The GRC team members received an informational letter about the content and participation in the study; this was voluntary. All data were processed anonymously, and privacy was respected according to the requirements of the Personal Data Protection Act. The data of patients from three months before and three months after the project were anonymously retrospectively extracted from the database of the digital patient file (DPF). The study was approved by the medical ethics committee of the Hanze University of Applied Sciences under number heac.2022.027.

### Measuring instruments

For the quantitative pretest (baseline) and posttest (at 15 months) assessments, the Extended Professional Identity Scale (EPIS) and the QuickScan Interprofessional Collaboration (QS) were administered. In addition, the quality of care for the GRC patients was retrospectively retrieved from the patient’s digital datafile using the health outcome ADL independence (measured by the Barthel Index (BI)) and the number of inpatient days. For the qualitative assessment, open-ended questions from the QS and multiprofessional team consultation (MPC) observations were used.

#### Interprofessional identity (IPI)

The EPIS is an instrument utilized by teams to identify aspects of IPI. It is suitable for measuring the effectiveness of actions on interprofessional motivation [13]. With the EPIS, three domains (belonging, interprofessional commitment, and interprofessional beliefs) are used to pose propositions with the request to rate them (1 = completely disagree, 2 = disagree, 3 = neutral/no opinion, 4 = agree or 5 = completely agree). Item scores are summed by subscale (domain) and, for the overall IPI (EPIS total), all items are summed and averaged. A higher score indicates positive attitude toward IPC with a maximum score of 20 per domain and 60 in total (see Additional file 1). The EPIS was distributed anonymously for the main researcher at the team level.

#### Team collaboration

The QS measures important aspects of team collaboration whereby team members can reflect on the collaboration within teams and use the results to optimize it. They subsequently translate the outcomes into, for example, a development or follow-up process of aspects that they want to improve. These can be included in a team reflection for which the results can be further explored together. In the QS, five statements are posed with the request to rate the degree of presence (1 = Not, 2 = Limited, 3 = Satisfactory, 4 = Strong or 5 = Excellent presence) and to indicate whether it is a development point for the team/network (see Additional file 2). The average score from “Not” to “Excellent present” on the 5 domains of the QS (see Table 1) indicates where the teams stand in terms of collaboration. A score of 5 is the maximum score; the lower the score, the more attention this domain should receive [19–22].

The QS concludes with a number of open-ended questions for which the professional reflects on their own observations and draws their own conclusions about collaboration in their team. These include “what I want to eliminate”, “what do I want to keep”, “what do I want to change”, and “what action should I/we take”. The QS questionnaire was distributed anonymously for the main researcher at the team level.

**Table 1:** The 5 domains of the QS

1. Shared Values In interprofessional collaboration (IPC), it is important that the members together have and use the same principles and core values, also known as shared values. The questions are about these shared principles and values. 2. Context The second domain is about context (including assignment from the organization, expertise, and background of team members). Its influence turns out to be very important for cooperation. If team members are aware of their context, this appears to positively influence the effectiveness of collaboration. The questions provide insight into the context in which a team/network operates. 3. Structure and organization. Effective IPC also appears to be related to how it is organized. Consider questions such as: “Have we made good agreements? Are we using the right working procedures? What is the division of roles in the team? Do we regularly evaluate our collaboration? 4. Group dynamics and interaction Interprofessional cooperation becomes more effective when the interrelationships between members are positive, there is an open atmosphere and group climate, and when feedback is provided, and critical interaction is possible. The questions in this domain deal with what happens in the group and in the interpersonal interaction. 5. Entrepreneurship and management The last domain is about the preconditions for effective cooperation. Consider questions such as: Is the team up to date with recent laws and regulations? Is attention paid to public relations (PR) (Do we, as a team, make ourselves known to the outside world?) and marketing? Is there a business plan, agreement, or something similar that defines everyone’s hours commitment and tasking other facilitation?	

#### Team communication

To properly understand the communication and behavior during the MPC team meetings, observations were made at three different levels of communication: 1) procedural level.

which includes content about how the team is working on their task, are procedures or methods used to achieve the goal, structure, and organization of the meeting; 2) content level associated with the topic and content of the meeting and what happens in the team, what information is exchanged by team members; and 3) interaction level concerning the team process and to what occurs between team members [19, 23](see Additional file 3). The MPC meetings were observed (pre and posttest) by two independent observers (main researcher and a physiotherapist) using a standardized observation list. The team was informed in advance of the observer’s arrival. We used the “fly on the wall (recognized outsider)” method which is an observation technique that allows the researcher to observe an environment without drawing the attention of the respondents [24]. The other observer was trained by the main researcher on the used observation list and method.

#### Quality of care (ADL and inpatient days)

Patient quality of care was measured by the number of inpatient days and the level of gained ADL independence of the patients cared for by the six GRC teams. Within GRC, improving activities of daily living (ADL) is an important outcome measure. (Partial) independence in ADL largely determines whether a patient can return home. When the improvement of the ADL takes place in a shorter period of time, the patient can go to his/her own familiar environment earlier. ADL independence was measured with the Barthel Index (BI), a valid and reliable measurement that assesses a patient’s ability to self-care [25]. Ten items related to the ADL and mobility are rated by the patient’s caregiver based on the amount of assistance needed to complete each activity. A higher score indicates more ADL independence with 20 being the maximum number of points. The BI is routinely administered at admission as well as at discharge and is registered in the DPF.

### Training program

The following collaborative actions on IPC education and team collaboration occurred between the pre and posttest measurements.

#### Team trainings

All GRC teams received two training sessions. The first was the certified team training “Turning the team on” [26] which began immediately after the pretest measurement. This training consisted of four days spread over four months with a focus on patient- and goal-oriented rehabilitation, the rehabilitation climate, IPC, and making improvement plans together. The second was the certified team training “Interprofessional Neurorehabilitation” provided by an external party [27]. It consisted of six one-day meetings aiming to increase the knowledge of neurorehabilitation and improve team collaboration with interprofessional work assignments and case discussions.

#### Webinars

The education for IPC further consisted of three webinars (online events) of 60 min; (1) an interactive webinar on how to collectively develop into an excellent rehabilitation team as part of the “Turning the team on” training [26], (2) an interactive webinar specifically on IPC, and (3) a recorded webinar on IPC presented on the organization’s own local digital intranet that was accessible to all professionals.

#### Online sessions

After the pretest online interprofessional team sessions (they were held online due to the applicable Covid-19 restrictions) were held for each team. During this session, each GRC team received feedback from the QS reflecting on their collaboration. The main points of feedback were discussed within the teams and processed into points for improvement and development under the supervision of a general project leader and the specialized geriatric nurse involved in each group.

### Statistical analysis

A conservative testing approach was applied assuming the pre and posttest team participants to be independent. Two-way analyses of variance (ANOVA) were employed to test for the differences in mean on the different outcomes. The EPIS and QS measurements were taken as an outcome at each of the two time points (pre and posttest). It is common in daily practice that some TS members work in two different teams. Because the EPIS and the QS measure the IPC within a particular team, the questionnaire was completed by the TS member involved per team where he/she works, and these observations were included in the analysis. For the difference in BI and number of inpatient days, the mean of the last three months of 2020 and first three months of 2022 (i.e. before and after the training program) were used. The BI at discharge (as a measure of care quality) and the difference between BI admission and discharge (delta BI) (as a measure of progression) were utilized for statistical analyses. In both models, the effect of time (pre vs. posttest) was statistically controlled for fixed effects of teams. A P-value < 0.05 is considered statically significant. Response and participant characteristics are presented with descriptive statistics. SPSS (IBM) version 28 for Mac was used.

### Qualitative analysis

We analyzed data from the open-ended QS questions and MPC observations with integrative thematic analysis[28]. In phase 1, the data-analyst (H.D.) got familiar with the data by reading the transcripts. Initial codes were generated in phase 2 highlighting relevant data. In phase 3, the researcher generated themes reflecting categories in the data. The themes were reviewed (phase 4), redefined and renamed (phase 5) by all authors. In phase 6, the themes were reported in an overview table.

#### Researcher characteristics

The main researcher (H.D) has a background in geriatric physiotherapy and is the science and research coordinator of the organization where this research took place. The MPC observations were done by the main researcher and another geriatric physiotherapist of the organization. Both were not involved as a team member in the observed MPC’s.

## Results

Six GRC teams from three locations participated, and a total of 768 patients were admitted during the study. Two teams at one location were unable to complete the team training“Turning the team on” before the posttest measurement due to Covid-19.

### Response and characteristics

The pretest EPIS and QS response was overall: 60.6% (n = 106), NS: 68.4% (n = 65, woman = 92.3%, age = 41.9(11.4)) and TS: 51.3% (n = 41, woman = 80.6%, age = 42.0(12.2)). Posttest the total response was: 54.2% (n = 84), NS: 42.0% (n = 34, female = 94.1%, age = 48.8(10.5)) and TS: 67.6% (n = 50, female = 74.0%, age = 44.5(12.3)). Data was representative of the team composition of a GRC team. Descriptive statistics of EPIS and QS measures in total, across teams, nursing- and treatment staff, and sex are presented in Table 2.

**Table 2:** Descriptive statistics of EPIS and QS measures in total, across teams, nursing- and treatment staff and sex

Pretest	Total	Team 1	Team 2	Team 3	Team 4	Team 5	Team 6	NS	TS	Woman	Man
N	106	18	21	20	22	13	12	65	41	90	16
Age	42.1 (11.6)	41.7 (12.6)	42.8 (11.8)	42.4 (11.5)	46.1 (10.9)	37.0 (12.1)	39.2 (10.6)	41.9 (11.4)	42.0 (12.2)	41.9 (11.3)	42.9 (13.2)
**EPIS**											
Belonging	17.0 (3.2)	17.7 (2.3)	17.8 (2.2)	17.3 (2.9)	16.0 (4.2)	17.1 (3.3)	15.9 (4.0)	16.5 (3.3)	17.7 (2.9)	17.0 (3.1)	17.6 (2.4)
Commitment	16.5 (3.4)	17.1 (2.8)	17.5 (2.7)	16.6 (3.1)	15.5 (3.9)	16.2 (4.6)	15.2 (3.9)	15.9 (3.6)	17.3 (3.0)	16.4 (3.5)	16.5 (3.1)
Believes	16.9 (3.0)	18.4 (1.7)	18.8 (1.9)	16.2 (2.5)	15.2 (3.2)	16.8 (3.8)	15.8 (4.0)	16.8 (3.3)	16.9 (2.5)	16.9 (3.2)	16.5 (2.2)
EPIS Total	50.6 (8.9)	53.0 (6.3)	53.7 (6.2)	50.1 (7.7)	47.9 (11.0)	50.1 (11.5)	46.9 (11.2)	49.7 (7.7)	51.8 (7.6)	50.1 (9.5)	50.7 (5.8)
**QS**											
Shared Values	3.2 (0.7)	3.4 (0.9)	3.6 (0.9)	2.9 (0.5)	3.1 (0.6)	3.0 (0.6)	3.2 (0.8)	3.4 (0.8)	2.9 (0.6)	3.2 (0.7)	3.3 (0.6)
Context	3.1 (0.7)	3.3 (0.7)	3.3 (0.7)	3.0 (0.8)	3.1 (0.6)	3.3 (0.7)	3.0 (0.6)	3.2 (0.7)	3.1 (0.6)	3.1 (0.7)	3.4 (0.6)
Structure / Organization	2.7 (0.7)	2.8 (0.9)	2.9 (0.9)	2.6 (0.6)	2.8 (0.5)	2.6 (0.7)	2.5 (0.5)	2.8 (0.7)	2.5 (0.7)	2.7 (0.7)	2.6 (0.7)
Group dynamics / Interaction	3.2 (0.7)	3.2 (0.8)	3.3 (0.8)	3.2 (0.8)	3.3 (0.8)	3.1 (0.6)	3.0 (0.5)	3.1 (0.7)	3.3 (0.8)	3.2 (0.7)	3.4 (1.0)
Entrepreneurship / Management	2.4 (0.8)	2.4 (0.8)	2.6 (0.8)	2.4 (0.9)	2.4 (0.7)	1.7 (0.6)	2.1 (0.8)	2.4 (0.8)	2.2 (0.8)	2.4 (0.8)	2.3 (0.9)
**Posttest**											
N	84	13	9	18	19	15	10	34	50	69	15
Age	43.5 (11.6)	46.4 (12.5)	44.3 (10.1)	46.2 (10.5)	44.4 (12.4)	38.4 (10.0)	40.7 (13.9)	41.9 (10.5)	44.5 (12.3)	43.6 (11.8)	42.7 (11.3)
**EPIS**											
Belonging	18.3 (2.0)	17.7 (1.9)	18.3 (1.9)	18.1 (3.0)	18.7 (1.5)	17.9 (1.8)	19.3 (1.0)	17.9 (1.8)	18.6 (2.1)	18.3 (2.1)	18.1 (1.7)
Commitment	17.4 (2.2)	16.5 (1.9)	16.4 (2.4)	17.5 (2.7)	18.0 (1.6)	16.9 (2.3)	18.8 (1.9)	17.1 (2.3)	17.6 (2.2)	17.5 (2.2)	16.7 (2.5)
Believes	17.5 (2.6)	17.5 (1.7)	17.4 (2.4)	17.5 (2.9)	17.5 (2.0)	16.7 (3.9)	18.8 (2.1)	17.4 (3.0)	17.6 (2.4)	17.5 (2.7)	17.2 (2.0)
EPIS Total	53.2 (5.6)	51.8 (4.4)	52.2 (5.7)	53.0 (7.7)	54.1 (3.6)	51.5 (5.7)	56.9 (4.2)	52.4 (5.4)	53.7 (5.7)	53.3 (5.7)	52.1 (5.0)
**QS**											
Shared Values	3.5 (0.6)	3.3 (0.6)	3.0 (0.4)	3.6 (0.6)	3.5 (0.6)	3.7 (0.8)	3.9 (0.6)	3.5 (0.7)	3.5 (0.6)	3.5 (0.6)	3.2 (0.4)
Context	3.4 (0.5)	3.3 (0.6)	3.2 (0.6)	3.4 (0.4)	3.4 (0.5)	3.5 (0.4)	3.7 (0.7)	3.4 (0.5)	3.4 (0.5)	3.4 (0.5)	3.5 (0.4)
Structure / Organization	3.0 (0.7)	3.1 (0.9)	2.7 (0.7)	2.9 (0.6)	2.9 (0.7)	3.1 (0.5)	3.7 (0.5)	3.0 (0.6)	3.1 (0.7)	3.0 (0.7)	3.1 (0.5)
Group dynamics / Interaction	3.6 (0.5)	3.5 (0.6)	3.6 (0.6)	3.5 (0.4)	3.5 (0.6)	3.5 (0.5)	3.9 (0.5)	3.4 (0.6)	3.6 (0.5)	3.5 (0.6)	3.6 (0.3)
Entrepreneurship / Management	2.6 (0.7)	2.6 (0.7)	2.4 (0.5)	2.5 (0.6)	2.4 (0.8)	2.7 (0.8)	3.1 (0.5)	2.5 (0.8)	2.6 (0.6)	2.6 (0.7)	2.3 (0.7)

EPIS = Extended Professional Identity Scale, QS = QuickScan Interprofessional Collaboration NS = Nursing staff, TS = Treatment staff

Data represent mean values (SD)

The number of patients admitted prior to the training program was 181 with an age (mean (SD)) of 80.2(10.4), woman = 56.9%. For the analyses, 102 discharge BI and 44 complete delta BI could be used. The number of patients admitted after the training program was 170 with an age of 80.9 (8.5), woman = 63.5%. For analyses, 91 discharge BI and 50 complete delta BI could be used.

### Interprofessional identity (IPI)

The two-way ANOVA show, after controlling for the team effects, a statistical significant training program effect of P = .001, 95%CI: 0.57–2.21 on “interprofessional belonging”, of P = .027, 95%CI: 0.12–1.90 on “interprofessional commitment”, and of P = .013, 95%CI: 0.62 − 5.20 on the EPIS total score. The “interprofessional beliefs” also improved though it was borderline statistically significant (P = .054, 95%CI: − 0.01–1.65) (Table 3).

**Table 3:** Effect of program (pre-posttest) on the EPIS domains controlled for team effects

	Belonging						
	B	Std. Error	t	P value	95% CI		
					Lower limit.	Upper limit	
Intercept	16.91	.65	26.07	< .001	15.63	18.19	
Training program	1.39	.42	3.33	.001	.57	2.21	
	**Commitment**						
Intercept	16.50	.70	23.45	< .001	15.12	17.88	
Training program	1.01	.45	2.23	.027	.12	1.90	
	**Beliefs**						
Intercept	16.89	.66	25.75	< .001	15.560	18.19	
Training program	.82	.42	1.94	.054	− .01	1.65	
	**Total**						
Intercept	50.45	1.81	27.93	< .001	46.88	54.01	
Training program	2.91	1.16	2.51	.013	.62	5.20	

Teams 1–6 = N.S. See Additional file 4 for detailed information on specific team effects

### Team collaboration

The two-way ANOVA analyses show, after controlling for the team effects, a statistical significant training program effect of P = .009, 95%CI: 0.07 − 0.47 on “shared values”, of P = .005, 95%CI: 0.08 − 0.44) on “context “, of P = .001, 95%CI: 0.14 − 0.56 on “structure and organization”, of P < .001, 95%CI: 0.18 − 0.58 on “group dynamics and interaction” and of P = .039, 95%CI: 0.01 −0 .48 on “entrepreneurship and management” (Table 4).

**Table 4:** Effect of training program (pre-posttest) on the QS domains after controlling for team effects

	Shared values					
	B	Std. Error	t	P value	95% CI	
					Lower limit.	Upper limit
Intercept	3.37	.16	21.34	< .001	3.06	3.68
Training program	.27	.10	2.63	.009	.07	.47
	**Context**					
Intercept	3.19	.14	23.58	< .001	2.93	3.46
Training program	.26	.09	2.82	.005	.08	.44
	**Structure and organization**					
Intercept	2.91	.16	18.60	< .001	2.60	3.22
Training program	.35	.11	3.30	.001	.14	.56
	**Group dynamics and interaction**					
Intercept	3.25	.15	21.62	< .001	2.95	3.54
Training program	.38	.10	3.75	< .001	.18	.58
	**Entrepreneurship and management**					
Intercept	2.45	.18	13.75	< .001	2.10	2.80
Training program	.25	.12	2.08	.039	.01	.48

Teams 1–6 = N.S. See Additional file 5 for detailed information on specific team effects

### Quality of care (ADL and inpatient days)

Before the training program, the discharge BI (mean (SD)) was 15.6 (3.7) points, and the delta BI was 5.5 (3.4) points. After it, the discharge BI was 15 (5.1) points, and the delta BI was 5.4 (3.9) points. The mean (SD) number of inpatient days before the training program was 84.5 (24.2) days and 72.3 (23.6) days after. The inpatient days showed a statistically significant decrease of 11.8 days (P < .001, 95%CI: -17.34 - − 6.31) after controlling for the team effects (Table 5). Differences in the BI over time were not statistically significant, indicating that the number of inpatient days was significantly reduced without compromising quality of care.

**Table 5:** Effect of time (pre-posttest) on inpatient days after controlling for team effects

	Inpatient days						
	B	Std. Error	t	P value	95% CI		
					Lower limit.		Upper limit
Intercept	88.59	4.58	19.33	< .001	79.57		97.60
Time	-11.82	2.80	-4.22	< .001	-17.34		-6.31

Teams 1–6 = N.S. See Additional file 6 for detailed information on team effects

### Qualitative results of the open questions of the QS

At the initiation of the study, 88 (83%) open QS questions were completed. At the follow-up measurement, the open questions of 52 (61.9%) QS were completed. From the data analysis we generated the following main themes; “desired eliminations based on team expertise”, “desired retention based on team expertise”, “desired changes based on team expertise” and “required actions based on team expertise”. Subthemes were generated based on the domains of the QS; “shared values”, “context”, “structure and organization”, “group dynamics and interaction” and “entrepreneurship and management”. The relevant comments of the pre and posttest are presented based on the main themes and, if applicable, in the subthemes in Table 6.

**Table 6:** Qualitative results of the open questions of the QS

Relevant comments and quotes Main themes				
*Subthemes*	**Desired eliminations**	Desired retention	**Desired changes**	**Required actions**
*Shared values*	**Pretest** The standard formulated goals/actions and insufficient focus on the patient wishes.	**Pretest** The wishes of the patient must be central. Working and communicating together in a shared DPF	**Pretest** Strengthen the shared values and propagate vision. That the team does not function as a unit. It was regularly stated that the patient should be more central with goals adapted to the individual wishes, and the family must be more involved. Approaching the patient from a more holistic view, pursuing the same goal together, clearer policy and clear agreements in the rehabilitation plan and more control for the patient in the rehabilitation process. *“Now it is often a standard plan, concrete goals are missing and are too general”, “What is important for the patient?”*, and *“We should talk more with the patient and talk less about the patient”.*	**Pretest** Clarity in shared values is being missed. Discussing what is the common mission/vision. Discussing the tension between the patient's wishes and financial constraints and other imposed demands. Take action to put the patient central, to involve them in the total rehabilitation process, and to work person centered. In doing so, better educate/inform the patient and family about the rehabilitation goals.
	**Posttest** -	**Posttest** Keeping the same goal in mind, creating independence for the patient The rehabilitation climate and the established more intensive contact with patient and family.	**Posttest** Points of attention included creating the rehabilitation plan interprofessional with the patient, making goals clear to the patient, and involving the family more. More structure in the MPC is still needed.	**Posttest** Further actions on expanding the rehabilitation climate across the department, a clearer rehabilitation plan and more pro-active involvement of the patient in the rehabilitation process. Continue to work on vision development. Improving the MPC.
*Context*			**Pretest** Team members need to know and make use of each other's qualities and expertise more and get a clearer picture of what is expected of each other. More clarity is needed about the expectations of a GRC team from the organization.	**Pretest** Indicate expectations and problems more clearly and investigate where collaboration can lead to quality improvement. There should also be more awareness about the social environment of the GRC team (facilities and organizations).
			**Posttest** Everyone should know each other’s expertise more.	**Posttest** Gain clarity in each other's expectations regarding MPC and collaboration
*Structure and* *organization*	**Pretest** Team meetings without a clear structure and inefficient MPC. Not reflecting on collaboration. Two teams specifically stated that they want to eliminate the unrest that prevailed in the teams at that time due to the changing team composition.		**Pretest** Using measuring instruments during the MPC should be used to objectify goals. In doing so, obtaining a better picture of the goal/sub-goals and which concrete actions are needed and working more towards activities and participation: *“What is important for the patient to be able to return home?”*. During the MPC, team members no longer want to discuss everything but only the main goals. Agreements within the MPC must be fulfilled.	**Pretest** Reflecting on the MPC and discuss questions such as: “Is our goal to get the patient home as soon as possible or should the patient wishes be our goal?”. Planning reflection moments as an interprofessional team. To make use of prognostic measuring instruments and of the measuring instruments that have already been administered before admission to the GRC department.
	**Posttest** Team meetings without a clear structure and inefficient MPC.		**Posttest** -	**Posttest** Continue to work on the joint responsibility
*Group dynamics and interaction*	**Pretest** Lack of unity between disciplines, the perceived difference between the NS and TS and the feeling that some disciplines are more important than others. Matters appointed were “working on islands”, “thinking in boxes”, “not looking beyond one's own discipline”. Everyone doing things in their own way, not always being collegial, not working together enough, and not working according to the agreements made. A solution was also mentioned; *“don't just name the negative, but look further into possible solutions, keep discussing”.*	**Pretest** The short lines between the disciplines. Matters appointed were good/nice/collegiate/open atmosphere, trust, safety, good cooperation, accessibility, respect, solidarity, involvement, each other's expertise, and social interaction. In general, there is an environment in which the team members feel safe and in which cooperation between all disciplines is self-evident.	**Pretest** More intensive cooperation, unity (mutual trust) between the NS and TS. Forming one team again and improving mutual contacts and collegiality. Providing more feedback, questioning each other more, and having critical conversations. Reflect on the collaboration within the team and between other disciplines. Team members also want more case discussions with multiple disciplines and more meetings with a social purpose.	**Pretest** Approaching each other more to make optimal use of each other's expertise/qualities, involve disciplines earlier in case of problems, tackle tasks together as a team, helping each other and being more actively involved. Improve the contact and cooperation between the disciplines. There should be actions to improve consultation and communication but also being critical and addressing each other. Creating an atmosphere where everyone feels heard and seen. Share positive feedback, not just the negative.
	**Posttest** The perceived difference between the NS and TS is sometimes still present.	**Posttest** The pretest points should be retained. However, also mentioned was being there for each other, interprofessional collaboration, looking beyond your own expertise, being able to spar with each other without prejudice, asking each other for support or using each other's expertise.	**Posttest** The following points were identified that still need attention: listen better to each other, continue to give constructive feedback, and working according to agreements that have been made with each other.	**Posttest** Continue to work on team building. Occasionally sit together as a team to discuss team performance, reflect, give each other feedback, and learn from each other.
*Entrepreneurship and management*			**Pretest** There is a need for insight into the financial background of the GRC team.	**Pretest** Improvements must be made in terms of entrepreneurship and business operations and to function more like a company without forfeiting personal attention
			**Posttest** -	**Posttest** -

### Qualitative results of the MPC observation

From the data analysis we generated the following themes; “communication procedure”, “communication content” and “team interaction”.

The standard MPC duration and frequency was sixty minutes, once every week. Four teams had a separate room for team meetings, two teams temporarily used the shared patient’s living room for this. The number of patients which were discussed ranged from four to eleven. The number of disciplines present ranged from six to twelve. All observed MPC’s had a chairperson and a joint rehabilitation plan. None of the patients were present during the MPC’s.

The most salient posttest observation on the theme “communication procedure” was that the MPC meetings in all of the teams were much more structured. We observed that the meetings started on time and that the time per patient was better monitored. We also observed that having a chairperson and clarity about that role added to the structure. Unlike the pretest observations where the role of the chairperson was not always clear and a management function was missing, the specialist nurse had evolved into the role of a chairperson. The chairperson in all of the teams was more in charge, maintained an overview, asked questions, and summarized the actions discussed. On the theme “communication content”, it was especially noticeable that during the pretest the goals were general predefined goals (e.g. going home, walking independently). During the posttest, the wishes of the client were taken into account more often and the goals were described more specifically (e.g. going home within three week, walking independently with a walker in and around the house). We also observed that more professionals in the team monitored the provisional discharge date and worked towards it. During the pretest the physician sporadically mentioned the date of discharge, while during the posttest the provisional discharge date was mentioned by the physiotherapist, occupational therapist and nursing staff and what was required for this. On the theme “team interaction”, the most noticeable posttest observation was that there was more interaction between the disciplines. Disciplines thought along with each other’s actions, gave advice, and consulted each other more. Decisions were made by the entire team instead of primarily by the physician. Reflection on the collaboration and insight into inpatient days had been introduced in two teams. The posttest observation on all theme’s showed that the active involvement of the NS had increased. They intervened in the discussions on their own initiative, brought in their expertise and what was important to the patient. For example, they indicated that they could also take over certain tasks of the physiotherapist, such as walking with the patient. Based on the three levels of communication, the relevant observations and whether they were present, absent, or not optimally present during the pre- and posttest measurements are shown in Table 7.

**Table 7:** Pre and posttest MPC observations based on the three communication levels

	Pretest	Posttest
**Procedure**		
The agenda was known in advance (invitation via e-mail)	V	V
A joint rehabilitation plan was used in an DPF with general goal, problem, action, and evaluation	V	V
The rehabilitation plan was projected on a screen thereby visible to those present	V	V
The rehabilitation plan was immediately updated by the physician or nurse practitioner	V	V
All disciplines also worked with the rehabilitation plan on their own tablet/laptop	V	V
Goals and actions were read at loud by the physician	V	V
A nurse specialized in geriatrics was the chairperson	V	V
The role of the chairperson was clear	V/X	V
**Content**		
The entire rehabilitation plan was discussed with each other	V	V
Goals and actions were stated by the physician and then the relevant discipline was asked how things were going with the patient and others supplemented this	V	V
Goals and actions were made specific	X	V
Concrete actions were discussed and immediately processed in the rehabilitation plan, including agreements for follow-up .	V	V
Actions and goals were jointly determined	X	X
The patients’ goals/preferences/values/needs were mentioned and were specifically stated in the joint rehabilitation plan	X	V/X
The patient was introduced and information about the diagnosis and background was provided.	V/X	V
Relevant problems and background information on the patient was discussed	X	V/X
A provisional discharge date was determined based on consensus.	V/X	V
The provisional discharge date was monitored by the entire team	X	V
Patient present at the MPC	X	X
The use of valid measuring instruments to support decision-making in the rehabilitation process	X	X
Discussion about the content of actions regarding treatment intensity, frequency, or agreements about joint actions	X	V/X
**Interaction**		
Disciplines communicated openly and there were short lines	V	V
Disciplines know and recognize each other and there was clear mutual respect	V	V
Equal involvement of all team members	X	V/X
Interaction between team members	X	V
Decision-making by entire team	X	V
MPC evaluation	X	X
Reflection on collaboration	X	V/X

V = present, X = not present, V/X = present, but needs improvement according to respondents

## Discussion

This study presents how GRC teams of a Dutch healthcare organization have developed IPC from the end of 2020 to the beginning of 2022. Consistent with the evidence obtained, there was a shift observed from mostly multiprofessional to more IPC. The improvement of the IPC resulted in a significant decrease in the number of inpatient days.

The initial EPIS scores already indicate that team members are motivated to work in an interprofessional team. This can be explained from the fact that professionals working in a GRC team are familiar with working with other professionals and have chosen in the past to do so. It is also possible that the EPIS results were initially overestimated. The concept and content of inter-professional versus multi-professional collaboration may not have been fully understood by all team members after all. Indications for this were ascertained during the first MPC observations. It was realized that, when the team members initially indicated that they were working together interprofessional, the authors observed a, albeit good, mainly multiprofessional collaboration. Despite this positive initial EPIS score, professional identity continued to improve throughout this study.

The QS shows average scores on team collaboration at the beginning of the study that indicate sufficient presence in the domains of “shared values,“ “context,“ and “group dynamics/interaction,“ but not on “structure/organization”, and “entrepreneurialism.“ The latter aspect is also difficult to achieve because it extensively focuses on a team’s entrepreneurship, and this plays less of a role in a nursing home setting. All domains have significantly improved statistically, however, on average, they still do not score strong and therefore continue to require attention. Progress being made on the aspect of “group dynamics/interaction” was also evident from the MPC observations. There is a clearer structure, more agreements are being kept, all disciplines are more actively involved, and there is more joint decision-making. There is an open atmosphere, team members know each other, and there is mutual respect. The basis for further developments towards interprofessional cooperation according to the meta-model of Reinders et al. is thereby present [15]. The teams began to improve accentuating the patient’s wishes/needs during the MPC; however, setting the rehabilitation goals more interprofessionally based on the patient’s wishes/needs still requires attention. The answers to the open QS questions demonstrate that the team members would also like to work on this issue.

The scores of the BI at discharge and the delta BI indicated no difference before and after the program, which is also in accordance with the expectations. Patients are discharged when they regain (some of) their ADL independence and thus have a higher BI score. The number of inpatient days was significantly reduced by 12 days. Progression in the patient’s rehabilitation as measured in delta BI is thus achieved in a shorter timeframe. An explanation for this significant decrease in inpatient days may be that, in addition to the shift to IPC, the MPC improved, and the discharge date was determined and monitored interprofessionally. Apparently other, often external, factors are important for discharge, and these are probably more quickly recognized by all professionals with IPC. The result is that rehabilitation is more streamlined and targeted towards discharge. Due to the applied design, this result must be interpreted with some caution, although a positive correlation of IPC with the length of stay has been described several times in literature [29].

This study was not focused directly on the effect of a particular training program but on following the development of the GRC teams toward the IPC in practice for one year. For the healthcare organization where this research took place, awareness, recognizing a common goal, and acknowledging everyone’s added value in the team was the primary goal. Nevertheless, research by van Dongen et al. in primary care teams does suggest that, when the team itself provides feedback and regularly reflects on its own group dynamics/interaction, organization, and structure of collaboration and team meetings, that this contributes to IPC [30, 31]. Moreover, there is evidence that improving team collaboration can contribute to effective communication, interpersonal relationships, and increased employee satisfaction [32]. Successful interventions for IPC should consist of 3 aspects: (1) interprofessional education, (2) interprofessional practice, and (3) interprofessional organization [18]. Retrospectively, it appears that the training program deployed by the organization incorporated these aspects. The education utilized various webinars and online team meetings as well as practice by working and reflecting on the points for improvement from the QS as a team. Aspects around organization, such as weekly MPCs in their own consultation room with joint insight into the rehabilitation plan were already present as this is inherent to working on a GRC team. It is unclear whether the webinars, online team meetings, and team reflection were used optimally during this study, however, personal communication indicates that this could have been better. In particular, the intense Corona period would have had a negative impact on this, because the teams were overloaded due to increased workload due to the absence of colleagues. Also it is possible that due to the personal impact of the pandemic, they may have been less willing or able to participate in the training program.

### Strengths and weaknesses of this study

A strength of this study is that it employs two measurements in time, showing the development of the teams in terms of the IPC. Additionally, the use of both qualitative and quantitative data allows for data triangulation ensuring less bias. The MPC observations and analyses of the qualitative data were performed by independent researchers. Using the QS questionnaire provides clear guidance for a team. A final strength is that there is consistency in the findings that point into the same direction towards improved IPC. The study also has several weaknesses. First, various actions were used to improve (interprofessional) collaboration, of which it is unclear which and to what extent they have contributed. Second, because the teams frequently change their composition, this study did not focus on the change within the individual professional. Third, to better interpret modifications in IPI and IPC, the use of in-depth interviews and/or focus group meetings would probably have provided additional useful information and is also a recommendation to include in the follow-up along with the results at the second QS measurement. Finally, Covid-19 most likely had an immense impact on both the actions deployed and the outcomes of this study, and the pandemic also placed many demands on the staff. Therefore, the fact that this response rate was ultimately achieved and that improvements have already been achieved is again positive.

### Implications for practice

If a true IPC is to be achieved within GRC teams, it is important to consider essential factors such as team collaboration, information sharing, and organizational factors. For the process towards IPC, it is advisable to delineate how a team is doing on these aspects. With the EPIS, the status of IPI can be quantified. The use of the QS proves to be a beneficial tool for assessing the quality of team collaboration and to gather input for reflection and optimization. This gives the team concrete tools with which they can work collectively. It is important to provide a common space for interprofessional consultation with a chairperson who monitors and summarizes the process thereby working in and from a joint rehabilitation plan for the proper exchange of information and considering the wishes and needs of the patient/family. It must subsequently be determined together who has the best qualities and competencies for certain roles in the team. It is recommended to first invest in becoming familiar with each other on a personal and professional level and then recognize that there is a common goal and that each other’s expertise is needed.

## Conclusions

Within the GRC teams of the healthcare organization, there has been a shift observed toward more IPC due to awareness and reflecting on team dynamics, organization, and structure of collaboration. There is also improvement on reflecting the wishes and needs of the patient by involving them more actively in the rehabilitation. ADL independence has not changed, yet we found a statistically significant decrease in the number of inpatient days. The basis for IPC is well established, however, it continues to be necessary that the teams continue to develop and invest in the collaboration with each other and the patient to further improve it.

### Electronic supplementary material

Below is the link to the electronic supplementary material.

Supplementary Material 1

Supplementary Material 2

Supplementary Material 3

Supplementary Material 4

Supplementary Material 5

Supplementary Material 6

## Data Availability

The datasets generated and/or analysed during the current study are not publicly available due to non-participant consent but are available from the corresponding author on reasonable request.

## List of Abbreviations

ADL: Activities of Daily Living
CI: Confidence Interval
DPF: Digital Patient File
EPIS: Extended interProfessional Identity Scale
GRC: Geriatric Rehabilitation Care
IPC: Interprofessional Collaboration
IPI: Interprofessional Identity
MPC: Multi-Professional team Consultation
NS: Nursing Staff
QS: QuickScan interprofessional collaboration
TS: Treatment Staff
