# Supplementary material for: Nursing home geriatric rehabilitation care and interprofessional collaboration; a practice-based study
Source: BMC Geriatr. 2023 Sep 5;23:539. doi: 10.1186/s12877-023-04212-6 (PMC10478267; doi:10.1186/s12877-023-04212-6)
Supplement: Supplementary file 1 — Supplementary Material 1 [file 12877_2023_4212_MOESM1_ESM.docx]

Additional file 1 EPIS

**EPIS**

Instruction

This questionnaire asks for your own opinion regarding *interprofessional collaboration* as a professional, teacher, student and/or researcher. There are no right or wrong answers possible.

*Definition*

*Interprofessional collaboration* occurs when professionals from different professional groups mutually complement and utilize each other to arrive at one optimal care path (or service) for each (complex) patient (or client). On the other hand, there is *multi-professional/ multi-disciplinary collaboration*, which only concerns delegation and referral. Where one reads healthcare professional, this may also concern other professional groups.

Your data will be processed carefully and anonymously. Thank you in advance for completing the questionnaire.

1 = completely disagree

2 = disagree **NB!**

3 = neutral / no opinion Please only give 1 answer or tick 1 box.

4 = agree If you want to change your answer, tick a new box and

5 = completely agree completely color the previous box.

__________________________________________________________________________________________

**General**

**Location / team**:……………………………………………….(multiple answers possible)

**Nursing staff / Treatment staff** (*Strike out what is not applicable*)

**Age:** ……………… *(in whole years)*  **Sex:**  male  female

_________________________________________________________________________________________

Extended Professional Identity Scale (EPIS)

**Interprofessional belonging**

1. I like meeting and getting to know people from other health

professions. 1 2 3 4 5

2. I feel a strong attachment toward interprofessional teams

comprising cross-disciplinary health professionals. 1 2 3 4 5

3. I like learning about other health professions 1 2 3 4 5

4. I enjoy learning and collaborating with people from other

health professions. 1 2 3 4 5

**Interprofessional commitment**

5. I would like to spend the rest of my career in an

interprofessional team 1 2 3 4 5

6. I prefer to see myself working in an interprofessional team 1 2 3 4 5

7. I identify myself with other members of an

interprofessional team 1 2 3 4 5

8. I am proud to be part of an interprofessional team 1 2 3 4 5

**Interprofessional beliefs**

9. Shared clinical decision-making should be an important part of

interprofessional collaboration 1 2 3 4 5

10. All members of an interprofessional team should be involved

in setting treatment goals for patients 1 2 3 4 5

11. Interprofessional team members should collectively agree

regarding patient care plans 1 2 3 4 5

12. When care decisions are made

interprofessional team members should strive for

consensus (agreement) on the planned processes 1 2 3 4 5

__________________________________________________________________________________________
