# Supplementary material for: Nursing home geriatric rehabilitation care and interprofessional collaboration; a practice-based study
Source: BMC Geriatr. 2023 Sep 5;23:539. doi: 10.1186/s12877-023-04212-6 (PMC10478267; doi:10.1186/s12877-023-04212-6)
Supplement: Supplementary file 2 — Supplementary Material 2 [file 12877_2023_4212_MOESM2_ESM.docx]

Additional file 2 QuickScan (QS)

Instruction

We have five domains, each with five statements that are important for effective (interprofessional team) collaboration. You score the statements on a scale of 1-5. In addition, for each statement you indicate whether you think this is (yes/no) a development point for the team. This is followed by some open questions in which you reflect on your observations and draw your own conclusions about the cooperation in your team/network.

Scoring:

You personally score all statements on the following format.

1= Not present (Gap, missing, lacks attention)

2= Limitedly present (Fragmented, not structurally embedded)

3= Sufficiently present (Practical in feasibility realized)

4= Strongly present (Sustainable and routine)

5= Excellent present (Best practice, exemplary, worthy of publication)

Development point:

If possible, write down a development point after scoring the question.

Yes = In my opinion, this is a development point for our team

No = Currently no development point

| **Score items Interprofessional Collaboration**  For person-centered care | **Scoring** | | | | | **Development point** |
| --- | --- | --- | --- | --- | --- | --- |
|  | 1 | 2 | 3 | 4 | 5 | Yes / No |
| **Shared Values** |  |  |  |  |  |  |
| 1. As a team, we carry a shared vision and mission. |  |  |  |  |  |  |
| 1. We provide person-centered care, where the goals   of the patient are central. |  |  |  |  |  |  |
| 1. We approach the patient from a holistic perspective. |  |  |  |  |  |  |
| 1. We create the preconditions for realizing 1 integrated care plan. |  |  |  |  |  |  |
| 1. We actively involve the patient and their environment in the total care process |  |  |  |  |  |  |
| **Context** | | | | | | |
| 1. We have a clear overview of the social map around our team (informal facilities, formal facilities and organizations). |  |  |  |  |  |  |
| 1. We know the interest and/or expectations of all team members for participation in team collaboration. |  |  |  |  |  |  |
| 1. We are familiar with the expertise of all disciplines and/or specialties involved in our team. |  |  |  |  |  |  |
| 1. We know each other's personal qualities within the team. |  |  |  |  |  |  |
| 1. We have a clear assignment (or expected result) from the organization |  |  |  |  |  |  |
| **Structure and organization** | | | | | | |
| 1. We adhere within the team to jointly established agreements (about content, procedures and dealing with each other). |  |  |  |  |  |  |
| 1. We work according to a clear structure during our team meetings (division of tasks and roles, agenda, working method, frequency). |  |  |  |  |  |  |
| 1. We use an unambiguous working method or methodology for joint discussion of patient cases. |  |  |  |  |  |  |
| 1. We regularly examine and reflect whether the team has the right composition (expertise, disciplines, qualities). |  |  |  |  |  |  |
| 1. We periodically reflect on the collaboration in our team. |  |  |  |  |  |  |
| **Group dynamics and interaction** | | | | | | |
| 1. We have a (conversation) leader (chairperson, team leader) who ensures effective collaboration. |  |  |  |  |  |  |
| 1. We give recognition to each participant (attendance, contribution, personal functioning). |  |  |  |  |  |  |
| 1. We have a pleasant group climate (mutual trust, us feeling, safe). |  |  |  |  |  |  |
| 1. We question and consult each other without bias. |  |  |  |  |  |  |
| 1. We have an open atmosphere in which we give each other feedback and can discuss disagreements, tensions or conflicts constructively. |  |  |  |  |  |  |
| **Entrepreneurship and business management** | | | | | | |
| 1. We are aware of trends, national developments, and policies (laws and regulations) that affect our team's collaboration. |  |  |  |  |  |  |
| 1. We pay attention to PR and marketing of our team. |  |  |  |  |  |  |
| 1. We have thought through legal implications of our team collaboration (legal entity, privacy laws, information sharing and storage, citizen consent). |  |  |  |  |  |  |
| 1. We have created a joint (business) plan in which everyone's hours of work and tasks are described. |  |  |  |  |  |  |
| 1. We have completed the essential preconditions (financial, personnel, equipment, ICT) to realize our mission. |  |  |  |  |  |  |

My conclusions:

After you complete the QuickScan, we ask you to reflect on the results and draw your own conclusions about collaboration in your team/network.

What do I want to eliminate:

What do I want to keep:

What do I want to change:

What do I / we need to take action on:
