# Supplementary material for: Nursing home geriatric rehabilitation care and interprofessional collaboration; a practice-based study
Source: BMC Geriatr. 2023 Sep 5;23:539. doi: 10.1186/s12877-023-04212-6 (PMC10478267; doi:10.1186/s12877-023-04212-6)
Supplement: Supplementary file 3 — Supplementary Material 3 [file 12877_2023_4212_MOESM3_ESM.docx]

Additional file 3. MPC observation list

OBSERVATION LIST MPC INTERPROFESSIONAL COOPERATION

| **Setting** |  |
| --- | --- |
| Location |  |
| Team |  |
| Date |  |

| **Setting variabels** |  |
| --- | --- |
| location |  |
| Duration (min.) |  |
| Frequency MPC |  |
| Number of attendees |  |
| Disciplines |  |
| Patiënt present? |  |
| Number of  Patiënts discussed |  |
| Chairperson present and who? |  |
| Common report? |  |

| **Observation (levels of communication)** |
| --- |
| **Procedure**  *How does the team work on their task; are procedures or methods used to achieve goal; structure and organization of the meeting.*  *Consider agenda, chairperson, time management, group size.* |
|  |
| **Content**  *Relates to the topic and content of the meeting and what happens in the team; what information is exchanged by team members.*  *Pay particular attention to naming the patients' background and health status, the professionals' questions to the team, whether or not the patients' goals are mentioned, the exchange of information, clarification of patients' values/preferences, concrete actions and decisions, and evaluation of the care plan.* |
|  |

| **Interaction**  *Relates to the team process and to what happens between team members.*  *Think short lines, mutual respect, knowing each other, working together constructively, wanting to learn, positive attitude.* |
| --- |
|  |
