# Supplementary material for: Nursing home geriatric rehabilitation care and interprofessional collaboration; a practice-based study
Source: BMC Geriatr. 2023 Sep 5;23:539. doi: 10.1186/s12877-023-04212-6 (PMC10478267; doi:10.1186/s12877-023-04212-6)
Supplement: Supplementary file 4 — Supplementary Material 4 [file 12877_2023_4212_MOESM4_ESM.docx]

Additional file 6. Effect of time (pre-posttest) on inpatient days after controlling for team effects

|  | **Inpatient days** | | | | | |
| --- | --- | --- | --- | --- | --- | --- |
|  | B | Std. Error | t | P value | 95% CI  Lower limit. Upper limit | |
| Intercept | 88.585 | 4.583 | 19.330 | <.001 | 79.566 | 97.604 |
| Pretest | -11.820 | 2.802 | -4.218 | <.001 | -17.335 | -6.305 |
| Posttest | 0 | . | . | . | . | . |
| Team1 | -.876 | 5.460 | -.160 | .873 | -11.620 | 9.869 |
| Team2 | -3.948 | 5.658 | -.698 | .486 | -15.083 | 7.187 |
| Team3 | -7.667 | 5.567 | -1.377 | .170 | -18.623 | 3.290 |
| Team4 | -10.755 | 5.514 | -1.950 | .052 | -21.607 | .098 |
| Team5 | .402 | 5.473 | .073 | .942 | -10.369 | 11.172 |
| Team6 | 0 | . | . | . | . | . |
| a. This parameter is set to zero because it is redundant. | | | | | | |
