# Supplementary material for: Nursing home geriatric rehabilitation care and interprofessional collaboration; a practice-based study
Source: BMC Geriatr. 2023 Sep 5;23:539. doi: 10.1186/s12877-023-04212-6 (PMC10478267; doi:10.1186/s12877-023-04212-6)
Supplement: Supplementary file 5 — Supplementary Material 5 [file 12877_2023_4212_MOESM5_ESM.docx]

Additional file 4. Effect of program (pre-posttest) on the EPIS domains controlled for team effects

|  | **Belonging** | | | | | |
| --- | --- | --- | --- | --- | --- | --- |
|  | B | Std. Error | t | P value | 95% CI  Lower limit. Upper limit | |
| Intercept | 16.907 | .648 | 26.074 | <.001 | 15.627 | 18.186 |
| Pretest | -1.387 | .416 | -3.333 | .001 | .566 | 2.208 |
| Posttest | 0^a^ | . | . | . | . | . |
| Team1 | .125 | .788 | .158 | .874 | -1.431 | 1.681 |
| Team2 | .644 | .797 | .808 | .420 | -.929 | 2.217 |
| Team3 | .132 | .762 | .173 | .863 | -1.373 | 1.637 |
| Team4 | -.306 | .752 | -.406 | .685 | -1.791 | 1.179 |
| Team5 | -.148 | .833 | -.178 | .859 | -1.793 | 1.496 |
| Team6 | 0^a^ | . | . | . | . | . |
|  | **Commitment** | | | | | |
| Intercept | 17.504 | .704 | 24.880 | <.001 | 16.116 | 18.893 |
| Pretest | -1.008 | .452 | -2.233 | .027 | -1.899 | -.117 |
| posttest | 0^a^ | . | . | . | . | . |
| Team1 | -.048 | .855 | -.056 | .956 | -1.736 | 1.641 |
| Team2 | .368 | .865 | .426 | .671 | -1.339 | 2.075 |
| Team3 | .041 | .827 | .049 | .961 | -1.592 | 1.674 |
| Team4 | -.325 | .816 | -.398 | .691 | -1.936 | 1.287 |
| Team5 | -.459 | .904 | -.508 | .612 | -2.244 | 1.325 |
| Team6 | 0^a^ | . | . | . | . | . |
|  | **Beliefs** | | | | | |
| Intercept | 17.709 | .656 | 26.997 | <.001 | 16.414 | 19.004 |
| Pretest | -.818 | .421 | -1.943 | .054 | -1.649 | .013 |
| Posttest | 0^a^ | . | . | . | . | . |
| Team1 | .798 | .798 | 1.001 | .318 | -.776 | 2.372 |
| Team2 | 1.030 | .806 | 1.277 | .203 | -.561 | 2.622 |
| Team3 | -.483 | .771 | -.626 | .532 | -2.005 | 1.039 |
| Team4 | -1.009 | .761 | -1.326 | .187 | -2.511 | .493 |
| Team5 | -.694 | .843 | -.823 | .411 | -2.357 | .970 |
| Team6 | 0^a^ | . | . | . | . | . |
|  | **Total** | | | | | |
| Intercept | 53.355 | 1.806 | 29.541 | <.001 | 49.790 | 56.919 |
| Pretest | -2.909 | 1.159 | -2.510 | .013 | -5.197 | -.622 |
| Posttest | 0^a^ | . | . | . | . | . |
| Team1 | .851 | 2.196 | .387 | .699 | -3.483 | 5.185 |
| Team2 | 1.982 | 2.221 | .893 | .373 | -2.401 | 6.364 |
| Team3 | -.350 | 2.124 | -.165 | .869 | -4.541 | 3.842 |
| Team4 | -1.005 | 2.096 | -.479 | .632 | -5.141 | 3.132 |
| Team5 | -1.264 | 2.321 | -.545 | .587 | -5.844 | 3.317 |
| Team6 | 0^a^ | . | . | . | . | . |
| a. This parameter is set to zero because it is redundant. | | | | | | |
