# Supplementary material for: Nursing home geriatric rehabilitation care and interprofessional collaboration; a practice-based study
Source: BMC Geriatr. 2023 Sep 5;23:539. doi: 10.1186/s12877-023-04212-6 (PMC10478267; doi:10.1186/s12877-023-04212-6)
Supplement: Supplementary file 6 — Supplementary Material 6 [file 12877_2023_4212_MOESM6_ESM.docx]

Additional file 5. Effect of training program (pre-posttest) on the QS domains after controlling for team effects

|  | **Shared values** | | | | | |
| --- | --- | --- | --- | --- | --- | --- |
|  | B | Std. Error | t | P value | 95% CI  Lower limit. Upper limit | |
| Intercept | 3.367 | .158 | 21.340 | <.001 | 3.055 | 3.678 |
| Pretest | .270 | .103 | 2.625 | .009 | .067 | .474 |
| Posttest | 0^a^ | . | . | . | . | . |
| Team1 | -.101 | .199 | -.510 | .611 | -.493 | .291 |
| Team2 | -.064 | .198 | -.325 | .746 | -.454 | .326 |
| Team3 | -.233 | .188 | -1.242 | .216 | -.604 | .137 |
| Team4 | -.204 | .184 | -1.106 | .270 | -.568 | .160 |
| Team5 | -.297 | .198 | -1.497 | .136 | -.689 | .095 |
| Team6 | 0^a^ | . | . | . | . | . |
|  | **Context** | | | | | |
| Intercept | 3.193 | .135 | 23.581 | <.001 | 2.925 | 3.460 |
| Pretest | .256 | .091 | 2.823 | .005 | .077 | .435 |
| posttest | 0^a^ | . | . | . | . | . |
| Team1 | -.030 | .174 | -.175 | .862 | -.373 | .313 |
| Team2 | .018 | .173 | .103 | .918 | -.323 | .359 |
| Team3 | -.146 | .162 | -.898 | .370 | -.465 | .174 |
| Team4 | -.087 | .160 | -.544 | .587 | -.402 | .228 |
| Team5 | .043 | .174 | .246 | .806 | -.301 | .386 |
| Team6 | 0^a^ | . | . | . | . | . |
|  | **Structure and organization** | | | | | |
| Intercept | 2.914 | .157 | 18.597 | <.001 | 2.604 | 3.223 |
| Pretest | .350 | .106 | 3.298 | .001 | .141 | .560 |
| Posttest | 0^a^ | . | . | . | . | . |
| Team1 | -.156 | .203 | -.769 | .443 | -.556 | .244 |
| Team2 | -.239 | .203 | -1.173 | .243 | -.640 | .163 |
| Team3 | -.343 | .187 | -1.829 | .069 | -.712 | .027 |
| Team4 | -.232 | .185 | -1.255 | .211 | -.597 | .133 |
| Team5 | -.215 | .199 | -1.081 | .281 | -.609 | .178 |
| Team6 | 0^a^ | . | . | . | . | . |
|  | **Group dynamics and interaction** | | | | | |
| Intercept | 3.247 | .150 | 21.617 | <.001 | 2.950 | 3.543 |
| Pretest | .377 | .101 | 3.747 | <.001 | .179 | .576 |
| Posttest | 0^a^ | . | . | . | . | . |
| Team1 | -.059 | .191 | -.310 | .757 | -.437 | .318 |
| Team2 | .043 | .190 | .227 | .821 | -.332 | .419 |
| Team3 | -.083 | .180 | -.463 | .644 | -.438 | .271 |
| Team4 | -.021 | .178 | -.117 | .907 | -.372 | .331 |
| Team5 | -.160 | .193 | -.829 | .408 | -.541 | .221 |
| Team6 | 0^a^ | . | . | . | . | . |
|  | **Entrepreneurship and management** | | | | | |
| Intercept | 2.449 | .178 | 13.749 | <.001 | 2.098 | 2.801 |
| Pretest | .246 | .118 | 2.081 | .039 | .013 | .479 |
| Posttest | 0^a^ | . | . | . | . | . |
| Team1 | -.068 | .224 | -.303 | .762 | -.511 | .375 |
| Team2 | .019 | .225 | .082 | .934 | -.426 | .463 |
| Team3 | -.078 | .212 | -.367 | .714 | -.497 | .341 |
| Team4 | -.183 | .210 | -.870 | .386 | -.598 | .232 |
| Team5 | -.318 | .231 | -1.377 | .170 | -.773 | .138 |
| Team6 | 0^a^ | . | . | . | . | . |
| a. This parameter is set to zero because it is redundant. | | | | | | |
